# Supplementary material for: COZOID: contact zone identifier for visual analysis of protein-protein interactions
Source: BMC Bioinformatics. 2018 Apr 6;19:125. doi: 10.1186/s12859-018-2113-6 (PMC5889581; doi:10.1186/s12859-018-2113-6)
Supplement: Supplementary file 2 — Software build. Executable binary file of the software tool. (ZIP 118,784 kb) [file 12859_2018_2113_MOESM2_ESM.zip › cozoid/platform/docs/testng-6.5.1-javadoc.zip!/org/testng/annotations/Configuration.html]

Configuration (TestNG)


---


|  |  |  |
| --- | --- | --- |
| **Overview** \| **Package** \| **Class** \| **Tree** \| **Deprecated** \| **Index** \| **Help** | |  |
| **PREV CLASS**   **NEXT CLASS** | **FRAMES**    **NO FRAMES**     **All Classes** |  |
| SUMMARY: REQUIRED \| OPTIONAL | DETAIL: ELEMENT |  |


---


## org.testng.annotations Annotation Type Configuration

---

**Deprecated.** *Use @BeforeSuite, @AfterSuite, @BeforeTest, @AfterTest,
\@BeforeGroups, @AfterGroups, @BeforeClass, @AfterClass, @BeforeMethod,
\@AfterMethod*

``` @Retention(value=RUNTIME) @Target(value=METHOD) public @interface Configuration ```

Configuration information for a TestNG class.

**Author:**
:   Cedric Beust, Apr 26, 2004

---

| **Optional Element Summary** | |
| --- | --- |
| `java.lang.String[]` | `afterGroups`             **Deprecated.** The list of groups that this configuration method will run after. |
| `boolean` | `afterSuite`             **Deprecated.** If true, the annotated method will be run after all tests in this suite have run. |
| `boolean` | `afterTest`             **Deprecated.** If true, the annotated method will be run after all every test. |
| `boolean` | `afterTestClass`             **Deprecated.** If true, the annotated method will be run after all the tests in the test class have been run. |
| `boolean` | `afterTestMethod`             **Deprecated.** If true, the annotated method will be run after any test method is invoked. |
| `boolean` | `alwaysRun`             **Deprecated.** For before methods (beforeSuite, beforeTest, beforeTestClass and beforeTestMethod, but not beforeGroups): If set to true, this configuration method will be run regardless of what groups it belongs to. |
| `java.lang.String[]` | `beforeGroups`             **Deprecated.** The list of groups that this configuration method will run before. |
| `boolean` | `beforeSuite`             **Deprecated.** If true, the annotated method will be run before this suite starts. |
| `boolean` | `beforeTest`             **Deprecated.** If true, the annotated method will be run before every test. |
| `boolean` | `beforeTestClass`             **Deprecated.** If true, the annotated method will be run after the test class is instantiated and before the test method is invoked. |
| `boolean` | `beforeTestMethod`             **Deprecated.** If true, the annotated method will be run before any test method is invoked. |
| `java.lang.String[]` | `dependsOnGroups`             **Deprecated.** The list of groups this method depends on. |
| `java.lang.String[]` | `dependsOnMethods`             **Deprecated.** The list of methods this method depends on. |
| `java.lang.String` | `description`             **Deprecated.** The description for this method. |
| `boolean` | `enabled`             **Deprecated.** Whether methods on this class/method are enabled. |
| `java.lang.String[]` | `groups`             **Deprecated.** The list of groups this class/method belongs to. |
| `boolean` | `inheritGroups`             **Deprecated.** If true, this @Configuration method will belong to groups specified in the @Test annotation on the class (if any). |
| `java.lang.String[]` | `parameters`             **Deprecated.** *Use @Parameters* |

### beforeTestClass

```
public abstract boolean beforeTestClass
```

:   **Deprecated.**:   If true, the annotated method will be run after the test class is instantiated
        and before the test method is invoked.

        :   **Default:**: false

---


### afterTestClass

```
public abstract boolean afterTestClass
```

:   **Deprecated.**:   If true, the annotated method will be run after all the tests in the test
        class have been run.

        :   **Default:**: false

---


### beforeTestMethod

```
public abstract boolean beforeTestMethod
```

:   **Deprecated.**:   If true, the annotated method will be run before any test method is invoked.

        :   **Default:**: false

---


### afterTestMethod

```
public abstract boolean afterTestMethod
```

:   **Deprecated.**:   If true, the annotated method will be run after any test method is invoked.

        :   **Default:**: false

---


### beforeSuite

```
public abstract boolean beforeSuite
```

:   **Deprecated.**:   If true, the annotated method will be run before this suite starts.

        :   **Default:**: false

---


### afterSuite

```
public abstract boolean afterSuite
```

:   **Deprecated.**:   If true, the annotated method will be run after all tests in this suite
        have run.

        :   **Default:**: false

---


### beforeTest

```
public abstract boolean beforeTest
```

:   **Deprecated.**:   If true, the annotated method will be run before every test.

        :   **Default:**: false

---


### afterTest

```
public abstract boolean afterTest
```

:   **Deprecated.**:   If true, the annotated method will be run after all every test.

        :   **Default:**: false

---


### beforeGroups

```
public abstract java.lang.String[] beforeGroups
```

:   **Deprecated.**:   The list of groups that this configuration method will run before.
        This method is guaranteed to run shortly before the first test method that
        belongs to any of these groups is invoked.

        :   **Default:**: {}

---


### afterGroups

```
public abstract java.lang.String[] afterGroups
```

:   **Deprecated.**:   The list of groups that this configuration method will run after.
        This method is guaranteed to run shortly after the last test method that
        belongs to any of these groups is invoked.

        :   **Default:**: {}

---


### parameters

```
@Deprecated
public abstract java.lang.String[] parameters
```

:   **Deprecated.** *Use @Parameters*

    :   The list of variables used to fill the parameters of this method.
        These variables must be defined in the property file.

        :   **Default:**: {}

---


### enabled

```
public abstract boolean enabled
```

:   **Deprecated.**:   Whether methods on this class/method are enabled.

        :   **Default:**: true

---


### groups

```
public abstract java.lang.String[] groups
```

:   **Deprecated.**:   The list of groups this class/method belongs to.

        :   **Default:**: {}

---


### dependsOnGroups

```
public abstract java.lang.String[] dependsOnGroups
```

:   **Deprecated.**:   The list of groups this method depends on. Every method
        member of one of these groups is guaranteed to have been
        invoked before this method. Furthermore, if any of these
        methods was not a SUCCESS, this test method will not be
        run and will be flagged as a SKIP.

        :   **Default:**: {}

---


### dependsOnMethods

```
public abstract java.lang.String[] dependsOnMethods
```

:   **Deprecated.**:   The list of methods this method depends on. There is no guarantee
        on the order on which the methods depended upon will be run, but you
        are guaranteed that all these methods will be run before the test method
        that contains this annotation is run. Furthermore, if any of these
        methods was not a SUCCESS, this test method will not be
        run and will be flagged as a SKIP.
        If some of these methods have been overloaded, all the overloaded
        versions will be run.

        :   **Default:**: {}

---


### alwaysRun

```
public abstract boolean alwaysRun
```

:   **Deprecated.**:   For before methods (beforeSuite, beforeTest, beforeTestClass and
        beforeTestMethod, but not beforeGroups):
        If set to true, this configuration method will be run
        regardless of what groups it belongs to.
          
        For after methods (afterSuite, afterClass, ...):
        If set to true, this configuration method will be run
        even if one or more methods invoked previously failed or
        was skipped.

        :   **Default:**: false

---


### inheritGroups

```
public abstract boolean inheritGroups
```

:   **Deprecated.**:   If true, this @Configuration method will belong to groups specified in the
        @Test annotation on the class (if any).

        :   **Default:**: true

---


### description

```
public abstract java.lang.String description
```

:   **Deprecated.**:   The description for this method. The string used will appear in the
        HTML report and also on standard output if verbose >= 2.

        :   **Default:**: ""


---


|  |  |  |
| --- | --- | --- |
| **Overview** \| **Package** \| **Class** \| **Tree** \| **Deprecated** \| **Index** \| **Help** | |  |
| **PREV CLASS**   **NEXT CLASS** | **FRAMES**    **NO FRAMES**     **All Classes** |  |
| SUMMARY: REQUIRED \| OPTIONAL | DETAIL: ELEMENT |  |


---
